# Congo Red Dot Paper Test for Antenatal Triage and Rapid Identification of Preeclampsia

**DOI:** 10.1016/j.eclinm.2019.02.004

**Published:** 2019-03-01

**Authors:** Kara M. Rood, Catalin S. Buhimschi, Theresa Dible, Shaylyn Webster, Guomao Zhao, Philip Samuels, Irina A. Buhimschi

**Affiliations:** a Department of Obstetrics and Gynecology, The Ohio State University College of Medicine, Columbus, OH 43210, USA; b Center for Perinatal Research, The Research Institute at Nationwide Children's Hospital, Columbus, OH 43215, USA; c Department of Pediatrics, The Ohio State University College of Medicine, Columbus, OH 43215, USA; d Department of Obstetrics & Gynecology, University of Illinois at Chicago College of Medicine, Chicago, Illinois, 60612, USA

**Keywords:** Preeclampsia, Cohort study, Congophilia, Misfolded proteins, Point-of-care

## Abstract

**Background:**

Proteins in the urine of women with preeclampsia (PE) bind Congo Red dye (urine congophilia). We sought to determine the diagnostic performance of a paper-based point-of-care test detecting urine congophilia for rapid triage and diagnosis of PE.

**Methods:**

Prospective cohort study conducted in 346 consecutive pregnant women evaluated for PE in the Labour and Delivery triage unit at our institution. The Congo Red Dot (CRD) Paper Test (index test) was performed on fresh urine samples. The CRD Paper Test results were compared to an expert adjudicated diagnosis in each case. The accuracy of the CRD Paper Test was also compared to urine and serum analytes (placental growth factor and soluble fms-like tyrosine kinase-1) previously proposed as diagnostic aids for PE.

**Findings:**

During the first triage visit, 32% (112/346) of women received a clinical diagnosis of PE. Yet, 63% (217/346) were admitted for in-patient diagnostic work-up or delivery. The CRD Paper Test was positive in 25% (86/346) of the cases. Adjudication confirmed PE in 28% (96/346) of all cases. The CRD Paper Test outperformed measured serum and urine markers (80·2% sensitivity, 89·2% specificity, 92·1% negative predictive value, 86·7% accuracy). The pre-test, positive and negative post-test probabilities were 27·7%, 74·0%, and 8·0%, respectively. Of women who were discharged undelivered, 38% (133/346) had at least one additional triage visit and the interval between the last negative and first positive CRD Paper Test was 12 (interquartile range, [5–34]) days.

**Interpretation:**

The CRD Paper Test is a simple, non-invasive, “sample-in/answer-out” point-of-care clinical tool for rapid identification of PE.

**Funding:**

Saving Lives at Birth Program and NICHD.

**Research in Context**

**Evidence Before the Study**

Preeclampsia (PE) remains a leading cause of maternal and perinatal morbidity and mortality worldwide. The World Health Organization estimates that 14% of maternal deaths in low-resource settings, approximately 341,000 cases per year, are caused by this disease. PE has a large spectrum of medical signs and symptoms resulting in a range of clinical phenotypes and outcomes, making a diagnosis on available clinical and laboratory parameters challenging. Hypertension and proteinuria are non-specific, and thus major challenges arise when differential diagnosis includes chronic hypertension, endocrine, and kidney diseases. Consequently, it is not uncommon when confronted with clinical ambiguity, particularly close to term, for physicians to indicate delivery even in the absence of a true diagnosis. During the last decade and despite major financial and research efforts, use of angiogenic factor tests for PE could not be implemented clinically considering their high costs and impracticality for point-of-care testing. Members of our group were the first to discover that PE is a protein conformational disorder, similar to Alzheimer's disease. We observed that proteins in the urine of preeclamptic women bind Congo Red dye, a feature called urine congophilia. To that end, our group reported that urine congophilia carries diagnostic potential for PE. However, previous research was focused on laboratory-based techniques and validation of congophilia. To our knowledge no studies have evaluated in a pragmatic framework the clinical utility of a simple, easy to use, non-invasive, low-cost, paper-based point-of-care test to diagnose PE within minutes, at the patient's bedside.

**Added Value of the Study**

This study adds value to the existing evidence by reporting for the first time in an unselected population the diagnostic characteristics of the Congo Red Dye (CRD) Paper Test for rapid diagnosis of PE, in a hospital's triage area. The CRD Paper Test has a high accuracy for diagnosis of PE and outperforms previously proposed serum and urine immunoassay tests as diagnostic aids for PE. Our analysis shows that it is not only inexpensive, easy to use, highly accepted by the nursing staff, but identifies women with PE within 3 min. If the CRD Paper Test results were available to obstetrical providers, a negative CRD Paper Test could improve wait times in obstetrical triage areas, avoid unnecessary admissions and lower the associated health care expenses. Furthermore, our findings have potential to improve accurate timing of patients' transfers to higher-acuity hospitals, and more targeted steroids and magnesium sulfate treatment in patients at risk of indicated preterm delivery from PE.

**Implications of All the Available Evidence**

Morbidity and mortality from PE are due to delay or misdiagnosis. Implementation of the CRD Paper Test in the triage area could be a useful tool for rapid diagnosis of PE, and avoidance of unnecessary deliveries. Further multi-center studies are warranted in high and low-income countries where CRD Paper Test has the potential to save thousands of lives.

Alt-text: Unlabelled Box

## 1 Introduction

Hypertensive disorders affect 15% of pregnancies and account for one quarter of the antenatal admissions [1]. Preeclampsia (PE) is a multisystem disorder specific to human pregnancy, and its incidence varies from 5 to 60% of gestations, depending on maternal co-morbidities [2], [3]. The low rate of maternal deaths in Western countries is in blunt contrast with the global setting where 70,000 women are estimated to die each year from PE [4], [5]. In the U.S., given the difficulty of predicting and diagnosing PE, especially in the presence of clinical confounders (e.g. hypertension, kidney disease, migraines), a significant proportion of pregnancy-related maternal mortality and morbidity is still attributable to PE [6], [7]. Hypertensive disorders are responsible for 2·6 million stillbirths occurring annually worldwide [8]. Because delivery of the baby is the only curative intervention for PE and avoidance of a stillbirth, iatrogenic prematurity will continue to be a challenge.

For the last decade, research has focused on exploration of different inflammatory and angiogenic biomarkers for diagnosing PE [9], [10], [11]. Our group discovered that PE women excrete urinary misfolded proteins, raising the prospect this hypertensive condition could be a protein conformational disorder [12]. We further discovered that urine of PE women exhibits congophilia which is the affinity of misfolded proteins for the azo-dye Congo Red (CR) [13]. Based on the above premises, we found that quantification of urine congophilia carries diagnostic and prognostic potential for PE [13]. Other groups validated the clinical usefulness of assessing congophilia in pregnant women for PE diagnosis using our research laboratory protocol on nitrocellulose [14], [15], [16].

Test turnaround times and avoiding unnecessary hospital admissions are increasingly important to clinicians faced with diagnostic uncertainty and increased health care costs. To address these gaps we modified our initial protocol for determination of urine congophilia in a manner that allows testing at the point-of-care level. Our hypothesis was that identification of urine congophilia by using a rapid diagnostic test will have high accuracy for diagnosis of PE at the patient's bed-side. We designed, developed and validated a simple bed-side, paper-based urine test kit, which we named the CR Dot (CRD) Paper Test. In this study our objective was to determine the diagnostic accuracy of the CRD Paper Test comparing it to the clinical diagnosis based on the full clinical workup of the women referred to Labour and Delivery (L&D) triage unit for evaluation of PE. The main outcome measure was Area Under the Receiver Operating Characteristic plot (AUROC) to confirm and rule out PE based on the adjudicated diagnosis.

## 2 Methods

### 2.1 Design, Study Setting, and Participants

346 consecutive pregnant patients were recruited in L&D triage unit at The Ohio State University Wexner Medical Center and followed prospectively until delivery from July 2014 to July 2015. Patients were referred from lower level healthcare facilities (local antenatal clinics or level II regional hospitals) for evaluation of hypertension and/or to rule-out PE. All women aged 18 years or older were eligible. Exclusion criteria were inability to provide informed consent and/or to establish accurate pregnancy dating based on last menstrual period confirmed by an ultrasound examination. All subjects provided written informed consent. The Human Investigation Committee of The Ohio State University Wexner Medical Center and of Nationwide Children's Hospital approved the study.

### 2.2 Study Protocol

Eligible, consenting women were approached for enrollment by certified research nurses immediately after their presentation to the triage area before initiation of the clinical work-up for PE. Of the approached women (n = 353), 98% agreed to participate. Patients were consented to provide a urine and matched venous blood sample. Refusal to provide a blood sample (n = 107) was not an exclusion criterion. Urine was collected in sterile containers and tested fresh without processing in the triage area. The result of the CRD Paper Test was read at 3 min. The patient and the clinical team were blinded to the results. All medical decisions were taken independent of our study protocol. Data collection was planned before initiation of the study and performance of the first CRD Paper Test. Further details about the study protocol and the processing of the remaining urine and blood samples are described in the appendix (p. 2).

Triage utilization was calculated as the time interval between patient check-in to release from the unit. Duration of hospitalization was monitored for all women admitted with uncertain PE status and discharged undelivered. The study protocol was registered using the ClinicalTrials.gov (NCT02455544) system.

### 2.3 Case Adjudication

Because incorrect classification of outcomes can lead to reduced power and biased estimation of the diagnostic performance of a test, the ability of the CRD Paper Test and of all the other researched biomarkers was made by comparing results to the adjudication of the triage diagnostic decision [17].

Each case was adjudicated by two independent board certified Maternal Foetal Medicine specialists blinded to the results of the CRD Paper Test (KMR & CSB). The ACOG Task Force definition of hypertensive disorders of pregnancy was applied [28]. Details are provided in the appendix (p. 2).

### 2.4 Assessment of Urine Congophilia and Analytical Validity of the CRD Paper Test Kit

**Fig. 1:**
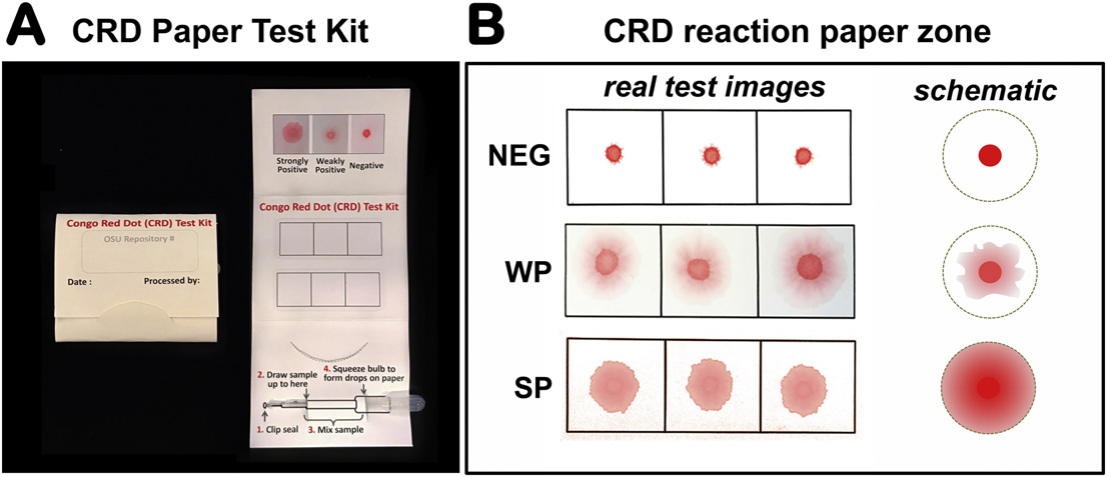
Congo Red Dot (CRD) Paper Test kits. A. Kits of the CRD Paper Test were manufactured “in house” by our research staff. Each kit had two label papers incorporated, and a visual colorimetric scale marked as strongly positive (SP), weak positive (WP) and negative (NEG). The kit contained a syringe prefilled with Congo Red dye. B. Representative images from three urine samples with NEG (n = 1), WP (n = 1), and SP (n = 1) CRD Paper Test results. Following application of the urine over the demarcated box the operator watches the test while it develops. Although the call is made at the end of the flow (~ 3 min), an impression of the result may be formed within the first few seconds from applying the sample. The scientific rationale behind the CRD Paper Test is presented in the appendix (p. 2).

The CRD Paper Test Kits were constructed in-house using Neenah Bright premium cardstock (Neenah Paper, Inc., Alpharetta, GA). Each kit contained printed instructions, a visual chromatic scale, a sealed pipette with standardized amount of CR dye and two reaction paper surfaces affixed to the mid-portion (Fig. 1A). Details about the rationale and procedure for the CRD Paper Test Kit are provided in the appendix (pp. 2–3). Briefly, for each patient, ~ 150 μL of fresh urine was mixed with the CR dye placed inside the transfer pipet. After ~ 1 min, the mixture was dispensed into approximately equal sized drops inside the areas printed on the reaction papers. The result was read at 3 min against the visual chromatic aid (Fig. 1B). The scientific principle behind the CRD Paper Test is described in the appendix (p. 2). Training for the user procedure and evaluation of the user acceptability for the CRD Paper Test are presented in the appendix (pp. 2–3). There were no indeterminate CRD Paper Test results, and no data were excluded for the final analysis.

The analytical validity of the CRD Paper Test kit was investigated in relation to the result of the CRD nitrocellulose array that was performed as described in the appendix (p. 6). The results of the CRD nitrocellulose array were reported as %CR Retention (%CRR) as previously published [13] (appendix, p 4). Although the nitrocellulose array is a simple method for a research laboratory, it was neither intended nor designed for point-of-care testing [13]. The technical validation analysis showed no difference between the two methods (appendix p 6).

### 2.5 Measurement of Additional Biochemical Markers

Levels of urine and serum soluble fms-like tyrosine kinase-1 (sFlt-1) and placental growth factor (PlGF) were immunoassayed as previously described [10]. The sFlt-1/PlGF ratios were calculated and used for comparison with the results of the CRD Paper Test; details provided in the appendix (p. 5). The test performances of the urine and serum sFlt-1/PlGF ratios were calculated based on optimal and previously reported cut-offs [10], [18].

### 2.6 Main Outcome Measure

The main outcome measure was the AUROC of the CRD Paper Test (index test) to confirm and rule-out PE based on the adjudicated diagnosis (reference standard). There were no missing data of the index test or reference standard.

### 2.7 Statistical Analysis

Statistical analyses were performed with Sigma Stat, version 2.03 (SPSS Inc., Chicago, IL) and MedCalc (Broekstraat, Belgium) statistical software. Normality testing was performed using the Shapiro–Wilk test. Data were compared with Mann–Whitney Rank Sum test, 1-way ANOVA followed by Holm–Sidak tests (parametric) or Kruskal–Wallis ANOVA on ranks followed by Dunn's tests (non-parametric). Immunoassay data was analyzed after logarithmic transformation. Spearman correlations were used to measure co-linearity between the selected independent variables. Comparisons between proportions were done with Chi-square tests.

ROC plots were used to determine optimal cut-offs for each test in our study population. Because most point-of-care tests intended for busy clinical settings have binary outcomes (positive/negative), these cut-off points were further used for calculation of diagnostic accuracy characteristics [19], [20]. Test accuracy (cases correctly classified/total number of cases), Youden Index [21], sensitivity, specificity, positive and negative predictive values, and likelihood ratios (LR) were calculated from contingency tables using both the optimal cut-off in our dataset and those previously published. We took this approach to facilitate comparisons with earlier results, but also to ensure our results are applicable to our current cohort where the prevalence of the disease could be different. Confidence intervals (CI) were calculated using the bootstrapping method. Graphical representations of ROC plots for dichotomised results were visualized using LRs and the Biggerstaff method [22]. AUROC was calculated both from the continuous data output and after conversion in LR coordinates as recommended for binary data [23]. Comparison of the of the index test to comparator tests was performed on the dichotomised data output using the non-parametric method of De Long. A *p* < 0.05 was considered significant throughout all analyses.

Pre-test probability was estimated based on the prevalence of a medically indicated delivery for PE (MIDPE) in our study population. The diagnostic utility of the CRD Paper Test was investigated using LR to estimate the post-test probability based on Bayes' theorem and Fagan's nomogram [24]. Our initial sample size calculation was performed to detect significance for an AUROC of 0.7 with an assumption of 3:1 ratio of negative to positive cases. 140 cases were estimated as necessary for error levels set at 0.05 and 260 cases for error set at 0.01. Our final sample of 346 cases was sufficient to detect significance in an AUROC of 0.630 compared to the null diagnostic value of 0.5. A *p* < 0·05 was considered significant throughout all analyses.

### 2.8 Role of the Funding Sources

The funding sources had no involvement in study design, data collection, analysis and interpretation, or writing the report. The corresponding author had full access to all the data and had final responsibility for the decision to submit the paper for publication.

## 3 Results

### 3.1 Patients and Associations Between Triage Diagnostic Decision, Adjudicated Diagnosis, and CRD Paper Test Result

Demographic and outcome characteristics of the patients enrolled in the study are presented in Table 1. In our cohort of women recruited in a tertiary care medical center, 48% of the enrolled patients were nulliparous, 66% were white, and 72% were obese class I or above. The distribution of GA at the first and last triage visit, and the number of triage visits per patient (range 1–8 visits) are shown in the appendix (p. 7). Women were most often referred because of co-morbidities [e.g. nephropathy, lupus, cholestasis, fetal growth restriction (FGR)] that did not allow establishment of a PE diagnosis based on clinical criteria alone. Concern for PE in the setting of chronic (crHTN) and gestational hypertension (gestHTN) followed in frequency. Overall, only 10% of the referrals were for potentially early-onset PE. The majority (n = 206, 62%) of the women in the cohort had a term delivery. The prevalence of MIDPE was 36% (121/333) and of a cesarean delivery 44% (148/333), with 70 (58%) of the women with a MIDPE delivered through cesarean.

**Table 1:** Characteristics of women enrolled in the triage cohort.

Characteristics at enrollment and during gestation	n = 346
Maternal age (years)	29 [25–33]
Parity	
0	167 (48%)
1	87 (25%)
2	44 (13%)
≥ 3	48 (14%)
Race/ethnicity of women	
White	226 (66%)
Black/African American	101 (29%)
Hispanic	8 (2%)
Other	11 (3%)
Weight (kg)	97 [80–113]
BMI	35 [30–42]
BMI categories	
Underweight (< 18·5)	0 (0%)
Normal weight (18·5–24·9)	21 (6%)
Overweight (25–29·9)	66 (19%)
Obese Class I (30–34·9)	82 (24%)
Obese Class II (35–40)	70 (20%)
Obese Class III (≥ 40)	97 (28%)
Not recorded	10 (3%)
Multiple gestation	14 (4%)
Gestational age at first triage visit (completed weeks)	
< 20 weeks	2 (1%)
20–24 weeks	7 (2%)
25–27 weeks	16 (5%)
28–31 weeks	55 (16%)
32–33 weeks	50 (14%)
34–36 weeks	106 (31%)
37–38 weeks	78 (22%)
≥ 39 weeks	32 (9%)
Referral diagnoses to confirm or rule out PE in the setting of:	
Chronic hypertension (crHTN)	50 (14%)
Gestational hypertension (gestHT)	71 (21%)
Preeclampsia without severe features (mPE)	38 (11%)
Preeclampsia with severe features (sPE)	48 (14%)
Superimposed PE (spPE)	27 (8%)
Other^a^	112 (32%)
Disposition after first triage visit:	
Admitted to hospital	217 (63%)
Discharged home	129 (37%)
Number of triage visits during index pregnancy	
1 visit	212 (61%)
2 visits	91 (26%)
3–8 visits	43 (13%)


Characteristics at delivery	n = 333^b^
Gestational age (completed weeks)	
< 34 weeks	56 (17%)
34–36 weeks	72 (22%)
37–38 weeks	115 (35%)
≥ 39 weeks	90 (27%)
Medically indicated early delivery for preeclampsia (MIDPE) (completed weeks)	
< 34 weeks	39 (12%)
34–36 weeks	43 (13%)
37–38 weeks	32 (10%)
≥ 39 weeks	7 (2%)
Cesarean delivery	148 (44%)

Data are median [interquartile range (IQR)] or n (%).

a Conditions that necessitated central laboratory work-up to rule-out PE (nephropathy, lupus, fetal growth restriction [FGR], cholestasis, etc.).

b The difference is accounted by 13 women who were lost to follow-up upon discharge from triage (n = 13, 3·75%).

**Fig. 2:**
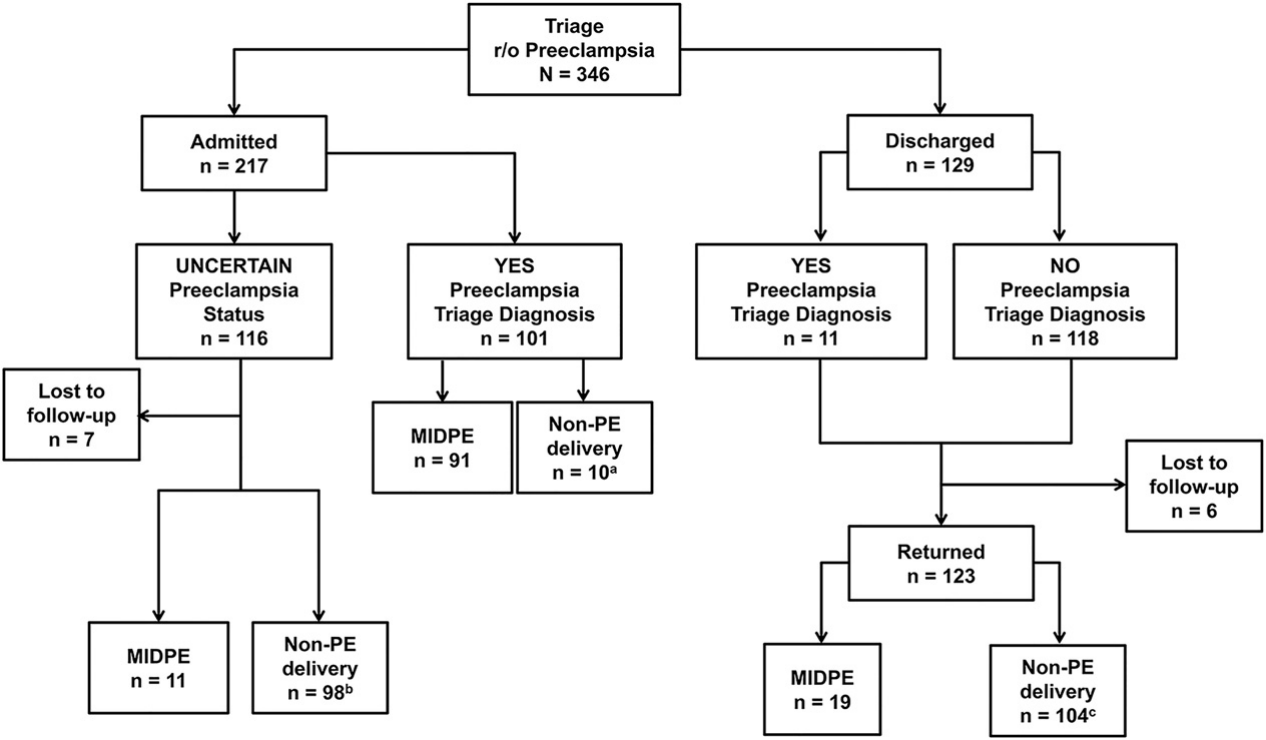
Consortium diagram of patient flow. We enrolled 346 patients. Hospital admission was recommended for 217 women (63%). Preeclampsia (PE) was the primary diagnosis for admission in 101 (47%) women. Of these, 91 (90%) had a medically indicated delivery for PE (MIDPE). In the remaining ten women (10%) delivery was indicated for non-PE indications (i.e. ruptured membranes, non-reassuring fetal status). Of the 217 admitted women, PE status was uncertain in 116 (53%) patients and required hospital in-patient workup. After in-hospital clinical and laboratory evaluation, 11 (9%) patients underwent a MIDPE. Of the women initially admitted with uncertain PE status, 98 (84%) had a preterm or term delivery unrelated to PE during the first or a subsequent admission. Out of all women enrolled, 129 (37%) patients were discharged after the initial triage visit. In 118 (91%), clinical and laboratory evaluation ruled-out (r/o) PE. The remaining 11 women (9%) were discharged with a diagnosis of PE with mild features, and a recommendation for outpatient follow-up. Of all discharged women, 123 (95%) returned to the hospital for a new triage visit. In the group who returned for at least one more triage visit, 19 (15%) ultimately had a MIDPE. Of women who returned and delivered at our hospital 104 (85%) did not develop PE. ^a^Indications for delivery (n = 10): non-reassuring fetal status n = 5; Preterm Premature Rupture of Membranes (PPROM) n = 2; chronic abruption n = 1; labour n = 1; cervical carcinoma n = 1. ^b^Indications for delivery (n = 98): spontaneous term labour n = 15; induction of labour at term (gestational age ≥ 37 weeks) n = 8; gestational hypertension n = 34; chronic hypertension n = 13; Intrauterine fetal demise n = 1; spontaneous preterm labour n = 6; chronic abruption/vaginal bleeding n = 1; proteinuria of unknown etiology n = 2; non-reassuring fetal status n = 11; PPROM n = 1; fetal growth restriction (FGR) n = 3; elevated liver functions tests (LFTs) n = 1; diabetes n = 1; induction for oligohydramnios n = 1. ^c^Indications for delivery (n = 104): spontaneous term labour n = 25; induction of labour at term (gestational age ≥ 37 weeks) n = 30; gestational hypertension n = 20; chronic hypertension n = 19; repeat cesarean for history of prior classical incision n = 3; spontaneous preterm labour n = 3; chronic abruption/vaginal bleeding n = 2; proteinuria of unknown etiology n = 1; PPROM n = 1.

A flowchart of the study population is presented in Fig. 2. Median triage utilization for discharged women was 191 [156–250] min. Of the patients admitted with uncertain PE status and who were discharged undelivered, the median duration of hospitalization was 2 (interquartile range (IQR) [1–3]) days. Of the patients enrolled in this study, 133 (38%) had more than one triage visit for PE evaluation.

**Fig. 3:**
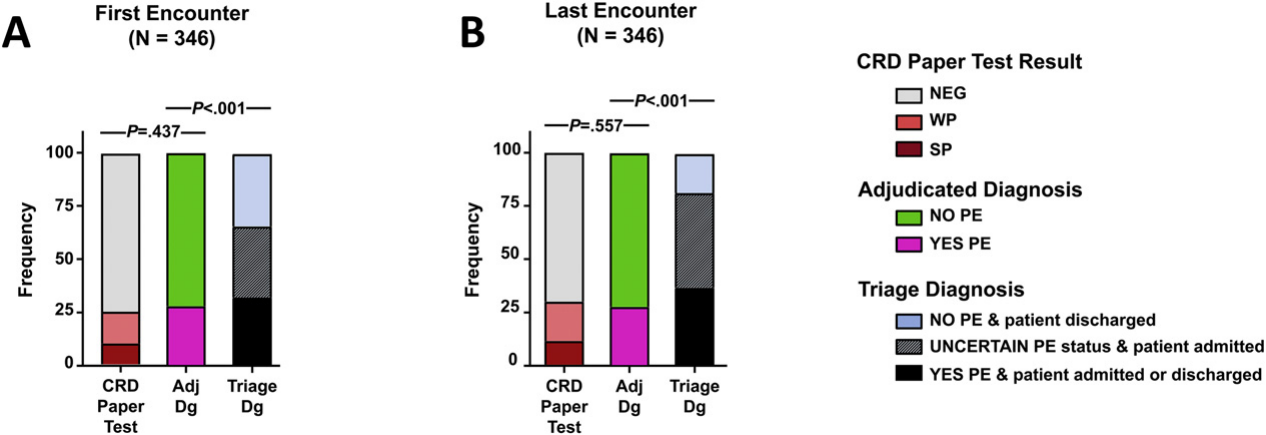
Proportions of positive and negative CRD Paper Test results based on clinical triage and adjudicated diagnoses. A. After the first triage visit, based on clinical criteria alone, 112 (32%) patients had preeclampsia (PE). However, after adjudication, only 92 (26%) patients met the diagnostic criteria. The adjudication process reclassified 20 (6%) patients. Clinical criteria for PE were not met in 18 patients. In this group just one patient had a weak positive (WP) Congo Red Dot (CRD) Paper Test result. Two patients initially considered to have uncontrolled chronic hypertension, were finally adjudicated to have super-imposed PE. Both women had WP congophilia. At the first encounter, 86 (25%) patients had a positive CRD Paper Test result. The prevalence of a positive CRD Paper Test result was non-significant compared to the prevalence of PE following case adjudication. Yet, based on clinical judgment alone, only 118 (34%) patients were discharged, with the majority of them admitted for either further PE work-up or for delivery. B. Similar relationships were seen based on the analysis of the last visit data. At the last triage encounter (proximal to delivery) 128 (37%) patients had a clinical diagnosis (Dg) of PE. Yet, based on adjudicated (Adj) diagnosis at the time of the last triage visit, only 96 (29%) patients had PE. Following the last triage visit 104 (30%) patients had a positive CRD Paper Test result. The only notable exception with data analysis based on the first visit was that the proportion of women (18%, 64) discharged based on ability to rule-out PE on clinical grounds alone was lower.

Urine congophilia was detected in 14 (12%) patients admitted with an uncertain diagnosis and in only 59 (58%) patients admitted with a diagnosis of PE established or confirmed in triage. There were 9 (8%) instances of positive congophilia in the group of patients discharged home absent PE. The CRD Paper Test was positive in just 4 (36%) patients discharged with a diagnosis of PE. Fig. 3 illustrates comparatively, the proportion of positive CRD Paper Tests, proportion of cases adjudicated as PE, and proportion of cases with a triage diagnosis of PE or admitted for further evaluation. In our study, 35 (10%) of the patients had at least one encounter where adjudication resulted in a change in diagnosis. No adverse events resulted from performance of the CRD Paper Test. The demographic and outcome characteristics of the patients grouped by the adjudicated diagnosis of the last triage visit are presented in Table 2.

**Table 2:** Characteristics of women grouped by adjudicated diagnosis.

Variable	NO preeclampsia adjudicated diagnosis n = 250	YES preeclampsia adjudicated diagnosis n = 96	p value
Maternal age (years)	30 [26–34]	28 [24–32]	0·041
Parity	1 [0–2]	0 [0–1]	0·114
Nulliparity	44 (46%)	52 (54%)	0·215
Multiple gestation	8 (3%)	6 (6%)	0·329
Race/ethnicity of women			0·851
White	166 (66%)	60 (63%)	
Black/African American	71 (28%)	30 (31%)	
Hispanic	6 (2%)	2 (2%)	
Other	7 (3%)	4 (4%)	
BMI (kg/m^2^)	35·4 [29·9–41·2]	34·5 [29·5–42·5]	0·968
Highest systolic blood pressure (mm Hg)	148 [137–163]	161 [145–177]	< 0·001
Highest diastolic blood pressure (mm Hg)	91 [82–99]	97 [86–106]	0·001
P:C ratio ordered	222 (89%)	84 (88%)	0·880
P:C ratio	0·2 [0·1–0·3]	1·0 [0·5–3·6]	< 0·001
P:C ratio ≥ 0·3	48 (22%)	74 (88%)	< 0·001
24 h proteinuria ordered	117 (47%)	63 (66%)	0·003
24 h proteinuria (mg/24 h)	244 [186–344]	810 [438–1923]	< 0·001
24 h proteinuria ≥ 300 mg/24 h	43 (37%)	59 (94%)	< 0·001
Gestational age at birth (completed weeks)*	38 [37–39]	34 [32–36]	< 0·001
Birthweight (grams)*	2068 [1485–2941]	3184 [2750–3567]	< 0·001
Cesarean delivery*	92 (39%)	56 (58%)	0·002
Newborn admitted to intensive care unit*	45 (19%)	60 (63%)	< 0·001
Maternal and/or fetal co-morbidities†			0·912
Nephropathy	7 (3%)	2 (2%)	
Lupus	3 (1%)	1 (1%)	
Chronic hypertension	76 (30%)	27 (28%)	
History of seizures	5 (2%)	2 (2%)	
History of migraines	26 (10%)	7 (7%)	
Cholestasis	2 (1%)	2 (2%)	
Fetal growth restriction	6 (2%)	5 (5%)	

Data are presented as median [interquartile range (IQR)] or n (%) and statistical comparison performed by Mann–Whitney or Chi-square or tests, respectively. *Excludes data from the 13 women lost to follow-up. †Some cases had more than one co-morbidity. The statistical comparison between groups is in proportion of cases with at least one of the listed co-morbidities.

Collectively, these data suggest that in a U.S. tertiary medical center patients that are referred to hospital triage for PE evaluation spend a long time in triage unit and a large number of them are admitted for further monitoring. Not all women discharged with a diagnosis of PE to be monitored as outpatients had a positive CRD Paper Test result.

### 3.2 Main Outcome Measure: Diagnostic Characteristics of the CRD Paper Test Result

**Fig. 4:**
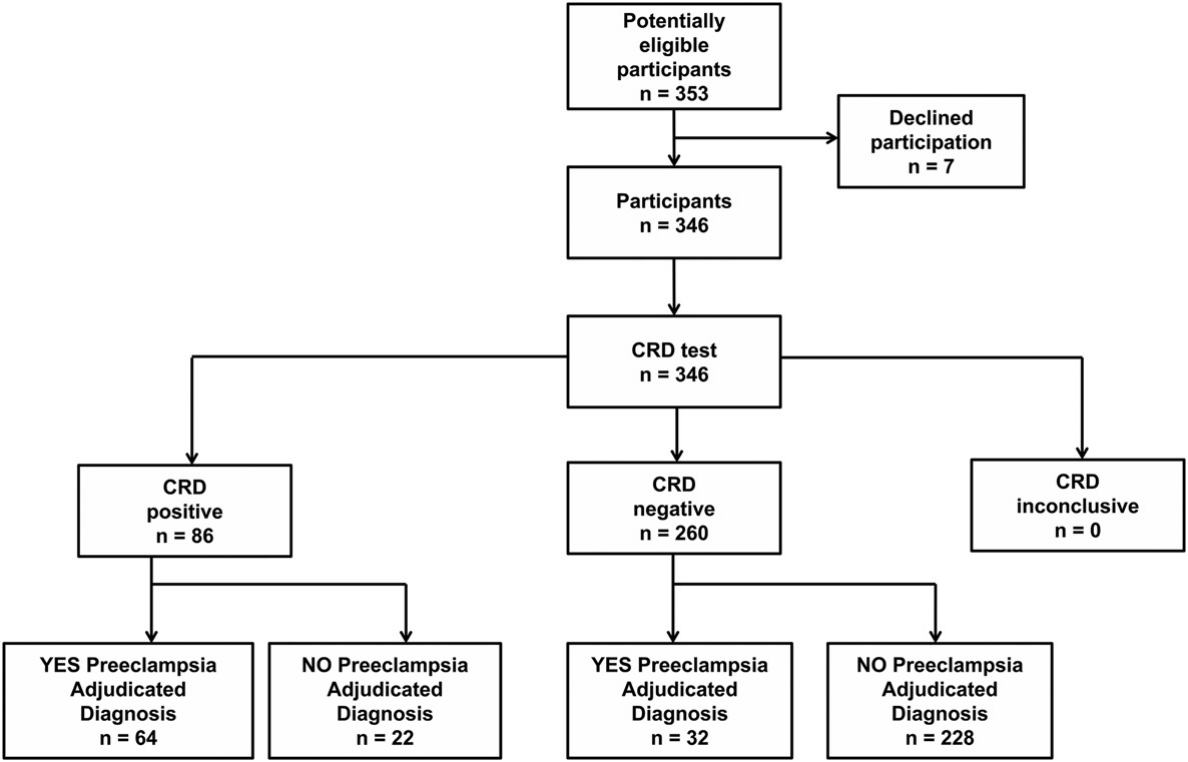
STARD flow diagram for a study of 346 enrolled patients undergoing triage evaluation for preeclampsia.

The breakdown of the population targeted for enrollment with the results of the CRD Paper Test based on the final adjudicated diagnosis is presented in Fig. 4. ROC analysis of the CRD Paper Test result on the ordinal scale (0–2) determined that the best model for PE diagnosis is achieved when WP = 1 and SP = 2 cases are grouped together. The main outcome measure, AUROC of the CRD Paper Test to confirm and rule-out PE based on the adjudicated diagnosis, was the highest compared to all other serum and urine biomarkers (Table 3). A positive CRD Paper Test (WP or SP) had 80·2% sensitivity, 89·2% specificity, 92·1% negative predictive value and 86·7% accuracy to correctly diagnose PE.

**Table 3:** Comparative accuracy results for predicting adjudicated PE diagnosis.

Test employed, n	AUROC [95% CI]	Cut-off	AUROC-LR [95% CI]	*p* value vs. CRD Paper Test	Sensitivity (%) [95% CI]	Specificity (%) [95% CI]	PPV (%) [95% CI]	NPV (%) [95% CI]	+ LR [95% CI]	− LR [95% CI]	Accuracy [95% CI]	Youden Index (*J*)
Urine												
CRD Paper Test, n = 346	0·850 [0·808–0·886]	WP or SP†	0·847 [0·805–0·883]	NA	80·2 [70·8–87·6]	89·2 [84·7–92·8]	74·0 [66·3–80·5]	92·1 [88·7–94·6]	7·43 [5·1–10·8]	0·22 [0·1–0·3]	86·7 [83·1–90·3]	0·694
Urine sFlt-1, n = 343*	0·723 [0·673–0·770]	> 29 pg/mL†	0·697 [0·645–0·745]	< 0.001	55·3 [44·7–65·6]	83·5 [78·3–87·9]	55·9 [47·6–63·9]	83·2 [79·7–86·2]	3·36 [2·4–4·7]	0·53 [0·4–0·7]	75·8 [71·3–80·3]	0·389
Urine PlGF, n = 343*	0·678 [0·625–0·727]	≤ 29 pg/mL†	0·666 [0·613–0·716]	< 0.001	64·9 [54·4–74·5]	68·3 [63·1–74·0]	43·6 [37·9–4·9]	83·7 [79·4–87·3]	2·05 [1·6–2·6]	0·51 [0·4–0·7]	67·3 [62·4–73·2]	0·336
uFP, n = 343*	0·765 [0·716–0·809]	> 1·7†	0·708 [0·657–0·756]	< 0.001	79·8 [70·2–87·4]	61·9 [55·5–67·9]	44·1 [39·5–48·8]	89·0 [84·3–92·5]	2·09 [1·7–2·5]	0·33 [0·2–0·5]	66·8 [61·8–71·7]	0·416
		> 2·1‡	0·699 [0·647–0·747]	< 0.001	60·6 [50·0–70·6]	79·1 [73·5–84·0]	52·3 [41·6–61·1]	84·2 [80·4–87·3]	2·90 [2·2–3·9]	0·50 [0·4–0·6]	74·1 [69·4–78·7]	0·398

Serum												
Serum sFlt-1, n = 239§	0·809 [0·753–0·856]	> 5300 pg/mL†	0·763 [0·704–0·816]	0.030	83·9 [71·2–92·2]	69·0 [61·8–75·6]	44·7 [34·9–54·8]	93·4 [87·8–96·9]	2·70 [2·1–3·5]	0·24 [0·1–0·4]	72·4 [66·7–78·1]	0·527
Serum PlGF, n = 239§	0·747 [0·687–0·801]	≤ 216 pg/mL†	0·700 [0·638–0·757]	< 0.001	80·0 [70·5–87·5]	52·7 [45·2–60·1]	35·6 [27·5–44·2]	93·3 [86·6–97·3]	1·85 [1·5–2·2]	0·24 [0·1–0·5]	60·7 [50·5–66·9·]	0·400
Serum sFlt-1/PlGF ratio, n = 239§	0·820 [0·765–0·866]	> 32·5†	0·757 [0·697–0·810]	< 0.001	81·8 [69·1–90·9]	69·6 [69·4–76·1]	44·6 [34·7–54·8]	92·8 [87·1–96·5]	2·69 [2·1–3·5]	0·26 [0·1–0·5]	72·4 [66·7–78·1]	0·514
		≥ 85, GA 20^0/7^–33^6/7^ ≥ 110, GA ≥ 34^0/7^‖	0·696 [0·633–0·753]	< 0.001	47·3 [33·7–61·2]	91·9 [86·9–95·4]	63·4 [46·9–77·9]	85·5 [79·6–90·0]	5·8 [3·3–10·1]	0·6 [0·4–0·7]	81·6 [76·7–86·5]	0·391

AUROC = area under the ROC plot. AUROC-LR = AUROC of the graph generated from likelihood ratio co-ordinates. PPV = positive predictive value. NPV = negative predictive value. + LR = positive likelihood ratio. − LR = negative likelihood ratio. CRD = Congo Red Dot. WP = weak positive. SP = strong positive. sFlt-1 = soluble fms-like tyrosine kinase-1. PlGF = placental growth factor. uFP = urine sFlt-1/PlGF. *Data missing for 3 patients due to insufficient sample volume. †Optimal cut-off in this population based on maximal Youden Index. ‡Previously published cut-off [10]. §Serum samples were not available for 107 women who did not agree to venipuncture. ‖Previously published cut-off [18]. Accuracy characteristics were analyzed based on samples collected at the last triage encounter.

LRs graphs comparing the CRD Paper Test to other urine and serum analytes dichotomised based on clinically relevant cut-offs are presented in the appendix (p. 8). For the subgroup of women where a 24-hour proteinuria was ordered and completed (n = 168), we compared the CRD Paper Test to blood pressures and total proteinuria as individual characteristics (appendix, p 9).

Using the prevalence of PE (27·7%) to estimate the pre-test probability in our population, the CRD Paper Test had a positive post-test probability of 74%, 95% CI [66–81] and a negative post-test probability of 8%, 95% CI [5–11] (appendix, p 10). The clinical interpretation of these results is that one in 1·4 patients with a positive test has PE, while one in 1·1 patients with a negative test does not have PE. Taken together, our results indicate that CRD Paper Test is an accurate and rapid diagnostic triage test for PE.

### 3.3 Breakdown Characteristics of Cases by MIDPE, Adjudicated Diagnosis and CRD Test Result

In Table 4 we present the grouping of cases based on final clinical outcome in real-life setting (MIDPE or no MIDPE), final adjudicated diagnosis and result of the CRD Paper Test. In the NO-MIDPE group 181 (90%) patients were adjudicated as non-PE and all had a negative CRD Paper Test. In the NO-MIDPE group, a positive CRD Paper Test was observed in 20 (10%) patients. Case by case analysis determined that these patients had a history of kidney diseases, crHTN or gestational hypertension (gestHTN). In the NO-MIDPE group 11 (5%) patients were adjudicated as PE. Of these, three (27%) patients had a negative CRD Paper Test, and were delivered for clinical indications other than PE. The other eight (73%) patients had a positive CRD Paper Test and were considered PE. However, following admission the primary indication for delivery was non-reassuring fetal status or PPROM.

**Table 4:** Breakdown of cases by outcome, final adjudicated diagnosis and Congo Red Dot (CRD) Paper Test result.

Clinical outcome^a^	Adjudicated diagnosis	Positive CRD test result	Case notes
NO MIDPE n = 212	NO PE n = 201	NO, n = 181 True negative	Unanimous concordance ruling out PE
		YES, n = 20 False positive	Cases in this category were medically indicated deliveries due to worsening chronic nephropathy, worsening crHTN or gestHT without a call of PE or spPE
	YES PE n = 11	NO, n = 3 False negative	Case adjudication was of PE w/o severe features or crHTN with spPE. All 3 cases were managed expectantly and all had medically indicated preterm deliveries for NRFHR or PPROM
		YES, n = 8 True positive	CRD concurred with the adjudicated diagnosis. All these cases were admitted following initial evaluation in triage (7 with a PE dg and 1 with uncertain PE status). Delivery indications were for all preterm for NRFHR or PPROM
YES MIDPE n = 121	NO PE n = 36	NO, n = 33 True negative	CRD concurred with the adjudicated diagnosis. Most MIDPE indications were in context of non-specific headache and the majority were near-term or at term
		YES, n = 3 False positive	Case adjudication was crHTN while the managing team's call was PE w/o severe features or spPE
	YES PE n = 85	NO, n = 16 False negative	Delivery indications and adjudication concurred as PE w/o severe features, spPE in the context of crHTN or HELLP syndrome features absent hypertension or proteinuria
		YES, n = 69 True positive	Unanimous concordance confirming PE

MIDPE = medically indicated delivery for preeclampsia. PE = preeclampsia. crHTN = chronic hypertension. gestHTN = gestational hypertension. spPE = superimposed PE. NRFHR = non-reassuring fetal heart rate. PPROM = preterm premature rupture of membranes. HELLP = hemolysis, elevated liver enzymes, low-platelet count.

a Table does not include the 13 cases lost to follow-up.

In the YES-MIDPE group 36 (30%) patients were adjudicated as NO-PE. In this subgroup, 33 (92%) patients had a negative CRD Paper Test. The majority of these patients were at term or near-term (GA: 36·8 [35·5–37·5] weeks), and delivery indications were most often prompted by non-specific headache. Among the three YES-MIDPE patients adjudicated as NO-PE but with a positive CRD Paper Test, the primary indication for delivery was PE or spPE. In this scenario the adjudicated diagnosis was crHTN. In the YES-MIDPE group, the adjudicated diagnosis was in agreement with the delivery indication in 85 (70%) patients. In this subgroup, 69 (81%) patients had a positive CRD Paper Test. Yet, 16 (19%) patients with YES-MIDPE who were adjudicated as having PE had a negative CRD Paper Test. This subgroup was populated by a heterogeneous mix of cases that had crHTN, isolated features of HELLP, absent hypertension or proteinuria or a spectrum of clinical symptoms non-specific for PE in patients with complex co-morbidities (i.e. headache in the context of history of migraines, epigastric pain in a patient with history of gastro-esophageal reflux). In summary, in both NO-MIDPE and YES-MIDPE groups most false positive or false negative CRD Paper Test results occurred in the contest of PE imitators such as crHTN, gestHTN, or kidney disease.

### 3.4 Characteristics of Cases With Multiple Triage Visits

Out of all patients enrolled 133 (38%) were referred to triage more than once. Select characteristics of cases grouped by the sequence of CRD Paper Test results are presented in the appendix (p. 5). In our cohort, CRD Paper Test was consistently negative in 88 (66%) patients. Of this group 3 (3%) patients were adjudicated as PE. Interestingly, 14 (16%) patients had a MIDPE at term. 22 (6%) patients with an initially negative CRD Paper test, displayed congophilia at a subsequent visit. The median interval from the last negative CRD test and the first positive result was 12 [5–34] days. Women who tested consistently positive had the shortest time to delivery and the lowest GA to delivery. Only 4 patients tested negative after a prior positive (all WP) CRD Paper Test result. To summarize, the CRD Paper Test result can turn positive within 2 weeks prior to clinical manifestation of PE.

## 4 Discussion

In this study we examined the performance of the CRD Paper Test to diagnose PE in women who presented to L&D triage unit with clinical symptoms and signs requiring diagnostic work-up for hypertension. In comparison to previously reported urine and serum biochemical markers, the CRD Paper Test was superior in both establishing and ruling-out PE. We also determined that a significant proportion of women sent to triage to be evaluated for PE are admitted with an uncertain diagnosis. Out of this group the majority of patients are ultimately discharged undelivered. A minority of cases are discharged from triage with a diagnosis of PE to be followed-up in an outpatient setup. Interestingly, although perceived by providers as PE, some of these women did not have a positive CRD Paper Test result. For both NO-MIDPE and YES-MIDPE groups, case adjudication suggested physicians miss or over diagnose PE.

Although extensively studied for diagnosis and prediction of PE, serum and urine PlGF and sFlt-1, alone or in combination with uterine artery Doppler ultrasound, did not gain clinical momentum [25], [26]. The invasive nature of blood sampling, reliance on expensive, lengthy central laboratory procedures and skilled personnel, and difficulty in interpreting results relative to GA intervals and from different platforms may have played a role [25], [27]. The aforementioned factors hinder practical implementation of testing for PE in the real-world and even more so in low-resourced countries where morbidity and mortality from PE and eclampsia are the highest [4]. The operational simplicity of the CRD Paper Test fulfills the current needs for a diagnostic tool to aid in the rapid assessment and triage of women with uncertain PE diagnosis.

Traditionally, PE is defined as new-onset hypertension and proteinuria after 20 weeks of gestation [28]. Some members of the obstetrical community seem to hold firm to the view that PE is easy to diagnose. This position is difficult to support based on our pragmatic study design. We found that physicians who are unaware of the CRD Paper Test results admit approximately a third of triaged patients due to diagnostic uncertainty. In this group, after extensive, lengthy and expensive inpatient work-up, only 9% of initially triaged women ultimately received a diagnosis of PE and had MIDPE. This is not surprising. PE is a heterogeneous unpredictable syndrome with a large spectrum of medical signs and symptoms resulting in a variety of clinical phenotypes and outcomes. In triage, clinicians take repeated blood pressure measurements and obtain information about headache, visual disturbances, chest pain, epigastric pain which none are specific to PE [25]. As it was determined from the demographic characteristics of our population the majority of the women presenting in triage are obese and already hypertensive. Because history and physical examination have limited accuracy in such population, and cannot be used alone for management decisions, the CRD Paper Test is uniquely positioned to increase the effectiveness of the triage process and possibly reduce health care costs [25]. Specifically, the CRD Paper Test has the potential to cut the need for triage referrals, decrease the turn-around time for diagnosis, and shorten the length of stay in obstetrical triage and antepartum units by eliminating unnecessary hospital admissions and/or early deliveries. A recent U.S. healthcare utilization analysis that included both maternal and neonatal costs estimated that in 2012 the incremental cost of deliveries was $2·18 billion for the first 12 months after delivery of a mother with PE and $1·15 billion for infants born to mothers with PE [29]. These calculations underscore the importance of an accurate test to diagnose PE.

Several groups have validated, in cohorts different than ours, that women with PE have elevated urine congophilia [14], [15], [16]. Importantly, all of these studies employed our previously published nitrocellulose laboratory protocol [13]. Most recently, Nagarajappa et al. concluded that urinary congophilia can be used to identify PE women from normotensive pregnant women [15]. Their study replicated our protocol in a laboratory hospital in rural India and showed that urinary congophilia was not affected by clinical variables such as GA of onset, severity, superimposition by eclampsia, fetal growth restriction or stillbirth. The authors opined that chronic kidney disorders (CKD) cannot be a major confounding factor in the clinical utility of urinary congophilia to diagnose PE as applied to the general pregnant population. In a prior study, McCarthy et al. confirmed elevated congophilia in PE, which was not present in non-pregnant women with systemic lupus erythematosus alone [16]. However, they noted some non-pregnant, advanced age women with lupus nephritis and some pregnant women with undefined CKD exhibited urine congophilia. This feature may reflect renal amyloidosis, a pre-Alzheimer state or PE in subclinical state for which they did not control or comment. Regardless, McCarthy and colleagues used urine samples from their highly selective Registry of Connective tissue diseases repository, which cannot be an accurate reflection of the general pregnant population [16]. We hope that the current study using the new CRD Paper Test will further enable and encourage other groups to perform studies for PE at point-of-care in different populations at high- and low-risk of PE.

The results of the HYPITAT-I trial changed physicians behaviour and attitude toward labour induction in women with hypertension at term [30], [31]. Analysis of the trend post-HYPITAT revealed that reflex delivery of a hypertensive woman at > 37 weeks led to an increase in inductions with decreased prevalence of PE [30]. Yet, what remains unknown is how many early-term (37–38 weeks) deliveries were indicated in the absence of PE, and what was the impact on neonatal outcomes, already known to be sub-optimal for early-term neonates [32]. Our adjudication process proved that ~ 30% of MIDPE cases did not meet full diagnostic criteria for PE. Most of these patients had a negative CRD Paper Test and were delivered at term for non-specific headaches. This approach is not unique, and emphasizes the tendency of U.S. physicians to more loosely opt for indicated delivery, especially approaching term. We believe that the CRD Paper Test has potential to shift the late preterm and early-term delivery curves to the right, and thus avoid unnecessary admissions to newborn critical care units [33]. Second, CRD Paper Test could be a useful tool to longitudinally monitor patients across gestation. In our study, the median time interval between conversion of a negative to a positive CRD Paper Test result was 12 days. Thus, the CRD Paper Test may help guide medical decision making regarding administration of steroids and magnesium for prevention of neonatal morbidity.

Clearly, not all women presenting at the hospital or in the ambulatory centers with PE-like symptoms should be delivered as they may not actually have PE but rather PE imitators [34]. The effectiveness of hypertension and proteinuria as diagnostic “gold standard” is even further compromised when PE is superimposed on conditions, such as crHTN, liver or chronic kidney diseases. The majority of false positives and false negative cases were observed in patients with crHTN, gestHTN and kidney disorder, where an accurate diagnosis of PE cannot be made on clinical grounds alone. However, it is important that clinicians make a correct diagnosis because the management and complications from these syndromes may differ. The CRD Paper Test adds clarity to help differentiate PE from PE imitators, which should result in fewer iatrogenic preterm deliveries.

Our pragmatic study design allowed us to evaluate patient flow in real-life practice conditions. Compared to prior studies that assessed immunoassay-based diagnostic devices in PE [35], our cohort evaluated the clinical utility of a point-of-care diagnostic tool in real time, and it was executed by clinical trained nurses in the triage area. Importantly, we had the ability to observe the patients longitudinally with maximal completeness of data and minimal lost for follow-up. The likelihood of discrepant interpretation of clinical and laboratory endpoints is increased in PE considering the diagnostic subjectivity and GA at evaluation. Our adjudication process allowed us to precisely point toward the cases where availability of the CRD Paper Test result would have potentially changed the clinical practice.

This study has several limitations, including recruitment at a single site. Although other groups already confirmed the value of congophilia [16], [14], this prospective cohort should be viewed as a key initial study to explore the significance of introducing the CRD Paper Test in the current clinical practice. Generalizability and cost effectiveness of using the CRD Paper Test in high and low income countries must be addressed. Such trials are currently ongoing. Reporting the results of a test modifies the clinical decision process vis-a-vis hospitalization of women with PE [36], [37]. By study design the physicians in charge were unaware of the results of the CRD Paper Test. Therefore, we could not calculate the financial impact communication of the results of the CRD Paper Test that could have had on healthcare system. We believe, reporting the results of the test to the practising physicians would have eliminated unnecessary hospital admissions, and many expenses including facility fees and the costs of laboratory testing and nursing care. Based on the number of patients discharged following their initial hospital admission, we can only approximate that at least 246 inpatient care days would have potentially been saved.

In summary, CRD Paper Test is an accurate, low technology, easy to use triage diagnostic tool that allows for accurate identification of PE within minutes.

## Authors' contribution

Drs. K.M. Rood, I.A. Buhimschi, and C.S. Buhimschi had full access to the data in the study and take responsibility for the integrity of the data and accuracy of the data analysis. Concept and study design: K.M. Rood, I.A. Buhimschi, C.S. Buhimschi; Acquisition, analysis, or interpretation of the data: K.M. Rood, T. Dible, S. Webster, G. Zhao, P. Samuels, I.A. Buhimschi, C.S. Buhimschi; Drafting of the manuscript: K.M Rood, I.A. Buhimschi, C.S. Buhimschi; Critical revision of the manuscript for important intellectual content: All authors; Statistical analysis: I.A. Buhimschi, C.S. Buhimschi, K.M. Rood; Obtained funding: I.A. Buhimschi, C.S. Buhimschi.

## Funding

Funded by Saving Lives at Birth (SLaB) Grant AID-OAA-A-14-00017 (IAB) from USAID, the Bill & Melinda Gates Foundation, Government of Norway, Grand Challenges Canada (Government of Canada), and the United Kingdom's Department for International Development (DFID). Additional funds were from National Institutes of Health (NIH) Eunice Kennedy Shriver National Institute of Child Health and Human Development R01 HD047321 (IAB).

## Declaration of Interests

IAB and CSB are named as inventors or co-inventors on patent applications filed by Yale University on the use of protein misfolding for diagnostic and treatment purposes of PE, some which are described in the article. Both received royalties from Yale University in accordance with institutional policies for inventorship. Commercial development of the CRD Paper Test has been licensed by Yale University to GestVision Inc. and stock awarded to IAB and CSB, in accordance with institutional licensing policies. A conflict of interest mitigation plan has been set in place at The Research Institute at Nationwide Children's Hospital and The Ohio State University as required for studies sponsored by federal funds. The other authors have no conflicts to declare.

## Appendix A Supplementary Data

Supplementary material Image 1
