# Supplementary material for: Congo Red Dot Paper Test for Antenatal Triage and Rapid Identification of Preeclampsia
Source: eClinicalMedicine. 2019 Mar 1;8:47–56. doi: 10.1016/j.eclinm.2019.02.004 (PMC6537515; doi:10.1016/j.eclinm.2019.02.004)
Supplement: Supplementary file 1 — Supplementary material [file mmc1.pdf]

CONGO RED DOT PAPER TEST FOR ANTENATAL TRIAGE AND RAPID IDENTIFICATION OF PREECLAMPSIA

Supplementary Appendix

Kara M. Rood, MD; Catalin S. Buhimschi MD; Theresa Dible RN;
Shaylyn Webster RN; Guomao Zhao, BSc; Philip Samuels MD;
Irina A. Buhimschi MD

Contents

1 Methods

1.1. Study protocol 2
1.2. Processing and storage of samples for laboratory-based analyses 2
1.3. Case adjudication protocol 2
1.4. How Congo Red Dot (CRD) Paper Test works 2
1.5. Rationale, development, training and procedure for the Congo Red Dot Paper Test 2
1.6. User acceptability survey 3
1.7. Laboratory procedure for the Congo Red Dot (CRD) nitrocellulose array 4
1.8. Laboratory procedures used to measure additional urine analytes 4

2 Supplementary Table

2.1. Breakdown of cases with at least two triage visits based on the sequence of the Congo Red Dot (CRD) Paper Test result (n=133) 5

3 Supplementary Figures

3.1. Analytical validation of the CRD Paper Test against the CRD nitrocellulose array 6
3.2. Distribution of gestational age and number of triage visits 7
3.3. Comparative likelihood ratio graphs of the CRD Paper Test against urine and serum markers measured in the research laboratory 8
3.4. Comparative likelihood ratio graphs of the CRD Paper Test against blood pressures and clinical measures of total proteinuria 9
3.5. Fagan plot analysis to evaluate the clinical utility of the CRD Paper Test based on pre- and post-test probabilities 10

4 References 11

1. Methods

1.1 Study protocol

Adherence to the study protocol was monitored by one of the investigators (KMR). Nurses collected information on maternal demographics, GA, medical and social history at enrollment. Maternal and neonatal outcome data were abstracted at discharge. If a patient was discharged undelivered, but readmitted to triage she was re-tested as a follow-up sample part of the study and not re-enrolled as a new case.

1.2 Processing and storage of samples for laboratory-based analyses

The container with any unused urine was transported to the research laboratory and a portion (5-10 mL) of urine transferred to a conical tube and centrifuged. Blood samples were collected by venipuncture at the time of urine collection using BD Vacutainer® Plus Plastic Serum Tube. Blood was allowed to clot. Urine and blood samples were spun at 3,000*g* at 4ºC for 20 min. Supernatants were collected and either analyzed or stored in aliquots at -80°C.

1.3 Case adjudication protocol

Cases were adjudicated based on all the clinical information that was documented during each encounter (clinical exam and laboratory results during each triage visit and/or in-hospital admission episode). If there was disagreement amongst the two adjudicators, the clinical case was discussed and a final diagnosis established. The adjudicated diagnosis of the last encounter prior to delivery was used as clinical gold standard. Case adjudication was dichotomic (PE present or absent). In cases without PE, an alternative diagnosis was noted.

1.4 How Congo Red Dot (CRD) Paper Test works

It is well known CR binds to cellulose fibers through hydrogen bonds with the cellulosic free hydroxyl (alcoholic) groups. Due to its planar molecular configuration, CR then intercalates between the fibers, hence the ability to stain textiles irreversibly. The same principle applies to misfolded proteins which due to the abundance of β-sheets have similar spatial arrangement as the cellulose fibers. When spotted on paper the free CR will form hydrogen bonds with cellulose, thus slowing down its flow through the porous paper surface (a phenomenon named by our group CR retardation). This explains the tight circle made by aqueous CR solutions on plain paper. If the sample contains aggregated proteins (e.g. PE urine), then a part of CR intercalates proportional with the concentration of the aggregates. When PE urine is placed on cellulose there will be only little or no free CR available for cellulose bonding. This explains why these aggregates spread forming a wide pink circle. A homogenously pink circle appears if all CR is engaged in amyloids. If the middle circle is still visible this means some free CR still exists available for bonding to cellulose. We tested systematically a wide range of papers with various cellulose properties and determined that self-adhesive labels represent the best cellulosic surface for application of the CR-urine solutions as these do not wrinkle when wet. This three-fold interaction between CR, aggregated proteins in PE urine and cellulose in the self-adhesive label paper led us to design the CRD Paper Test with components provided either in bulk or as kit assemblies.

1.5 Rationale, development, training, and procedure for the Congo Red Dot (CRD) Paper Test

The CRD Paper Test Kit was designed to contain all the materials required for a single test and to eliminate the washing steps. CRD Simple Test Kits were assembled weekly in our research laboratory at The Research Institute using materials commercially available during the study period. The CRD Simple Kit was developed and designed by one of the investigators (IAB) and is essentially a tri-folded piece of cardstock (Neenah Paper, Inc. Alpharetta, GA) on which information has been printed with laser colour printer.[1,2] Two different reaction papers were affixed to the cardstock's middle section (Avery 5160^{R} white easy peel laser address labels and Avery 6460^{R} white laser/ink jet removable multipurpose labels). Five µL Congo Red (CR) solution (Congo Red solution 0.5% aqueous, ScholAR Chemistry West Henrietta, NY, Cat# 9449604) was added to a fine tip 0.3 mL small bulb disposable transfer pipet (Samco Scientific). The end of the transfer pipet was then sealed using heat.

Prior to initiation of recruitment in the triage prospective cohort, clinically-trained research nurses were instructed on components of the CRD Paper Test Kit, technical procedure and trained to run the test and use the visual chromatic scale to score the final test result as negative (NEG=0), weak positive (WP=1), or strong positive (SP=2).

For this cohort, clinically trained certified research nurses who were not involved in patient management tested the urine samples. This was done in a utility room adjacent to the triage room so that the test development and result was visible only to the research nurse and not to the clinical team or patient. Following enrollment and collection of urine specimen, 150µL fresh urine was drawn up from the collection cup into the transfer pipet (after removal of the seal

using a nail clipper). Urine was mixed with the CR solution inside the transfer pipet; after 1 minute (described in the printed instruction) after which mixture was applied as 6 drops to the two different labels. Results were marked after 3 minutes on the visual aid printed inside the kit as NEG, WP or SP. For the purpose of calculating sensitivity and specificity for this prospective cohort both WP and SP results were interpreted as a positive CRD test result. For calculating correlations with the result of the CRD nitrocellulose array, an ordinal scale (0–2) was used: NEG=0, WP=1 and SP=2.

1.6 User acceptability survey

Survey: A questionnaire was developed to assess the nurses' acceptability of the CRD Paper Test Kit and its ease of use. The survey was administrated to each nurse at the end of their training period and. Each of the 7 recruiting nurses scored the CRD Paper Test with a score of 5 (very easy). The inter- and intra-operator variability in scoring the CRD Paper Test during the training period was <1%.

Questionnaire for Staff Conducting Point-of-Care Congo Red Dot (CRD) Test

Please take a few minutes to read through this questionnaire prior to preforming the Congo Red Dot (CRD) Point of Care Test. After you have run the test several times and you are familiar with it, please complete this questionnaire. Each research nurse conducting this test as part of the (CRD) test clinical trial should complete this questionnaire.

For approximately how many years (or months) have you been a research nurse? ________

For approximately how many years (or months) have you been a labor and delivery nurse? _______

How many specimens have you run on the new test? __________

Questions About CRD Test

How would you rate the ease of use in collecting specimens? (circle one)

Collecting specimens was….				
Very Difficult<br>1	Difficult<br>2	Neutral<br>3	Easy<br>4	Very Easy<br>5

How would you rate the package insert for ease of reading and understanding? (circle one)

The package insert was…				
Very Difficult<br>1	Difficult<br>2	Neutral<br>3	Easy<br>4	Very Easy<br>5

How would you rate the ease of use in running the test? (circle one)

Running the test was…				
Very Difficult<br>1	Difficult<br>2	Neutral<br>3	Easy<br>4	Very Easy<br>5

How would you rate the ease of reading the test result? (circle one)

Reading the test was…				
Very Difficult<br>1	Difficult<br>2	Neutral<br>3	Easy<br>4	Very Easy<br>5

How would you rate the read time for the test? (Check one)

- ☐ About right
- ☐ Too long
- ☐ Too short
- ☐ Don’t know
- ☐ Other: ________________________

What did you like best about the CRD Test?

____

____

What did you like least about the CRD Test?

____

____

What challenges do you see in using the CRD test as a point of care test in triage?

____

____

1.7 Laboratory procedure for the Congo Red Dot (CRD) nitrocellulose array

To confirm proof of principle feasibility of assessing urine congophilia using of our in-house assembled CRD Paper Test Kits, we conducted a side-by-side comparison of the paper-based test result and %Congo Red Retention (%CRR) measured using the CRD nitrocellulose array. The protocol for the CRD test on nitrocellulose along with the calculation of %CRR and the improvements that facilitate an automated image analysis were previously published.[3,4] Because the intended sample for the CRD Paper Test is crude urine we tested normalised and protein-normalised samples on the CRD nitrocellulose array. The protein-normalised result was used as technical "gold standard".

Briefly, 100 μL of protein-normalised or non-normalised urine are mixed with 2 μL of stock aqueous solution of Congo Red (CR, 5 mg/mL, Sigma Cat#C6277). A blank sample (BLK) is prepared by adding 2 μL of CR stock solution to 100 μL phosphate buffered saline (PBS). The urine-CR mixtures are vortexed for 1 h after which 5 μL of each mixture are spotted in duplicate onto a nitrocellulose membrane (Pure Nitrocellulose Unsupported Transfer membrane 0.22 μm, BioRad) that was pre-sized and punched in 3 of the 4 corners as previously reported. With the aid of a transilluminator the spots can be easily spaced to match the array format of our published stencil.[4] Each array holds a maximum of 84 spots thus allowing for samples from 41 patients to be batch-tested at a time. After spotting of samples, the array is left to air-dry (~15 minutes). The array in then rinsed with water for 3 minutes followed by increasing concentrations of methanol [50% methanol: 3 minutes, 70% methanol: 1 minute, 90% methanol until the redness in the BLK samples disappears completely (10-15 minutes). During this time, the red colour of non-PE samples fades as the free CR dye washes away. If urine contains misfolded proteins, the CR is retained by the misfolded proteins immobilised on the array and the spots remain visibly red. Images were taken before and after the methanol wash and converted to grayscale. %CRR was calculated as the % luminosity of each spot after the wash (nominator) from the luminosity before the wash (denominator). As a technical note is the recommendation in all our publications to use unsupported nitrocellulose (which does not contain cellulose acetate) as this feature is critical to assure complete washing of any free CR from BLK and urine samples. Presence of impurities in nitrocellulose membranes will result in high BLK values (%CRR>10%). For this publication, the quantification of the %CRR was done using the previously published smartphone-based image analysis algorithm updated to allow implementation on an iPod touch (Model 1574, Apple).[4]

1.8 Laboratory procedures used to measure additional urine analytes

The immunoassays were performed in one batch after all samples have been collected. Urine samples were analyzed for concentrations of placental growth factor (PlGF) and soluble fms-like tyrosine kinase-1 (sFlt-1) using commercially available ELISA assays (R&D Systems, Minneapolis, MN) in accordance to manufacturer's instructions. Previously stored urine and serum samples were thawed on ice and assayed in duplicate in a 96-well plate pre-coated with a capture antibody directed against free sFlt-1 or against PlGF. Incubation and washing protocols were performed, followed by reading at 450 nm with background subtraction. Minimal detectable concentrations in the assays for sFlt-1 and PlGF were 5 and 7 pg/mL, respectively. Data were reported and plotted with the Softmax software Pro 3.1.1 (Molecular Devices). Previously frozen urine supernatants were thawed at the same time, vortexed and 100 μL volume applied to each well without dilution. All samples measuring above the highest standard were re-assayed with 1:10 dilution. The urine sFlt-1/PlGF (uFP) ratio was calculated as previously reported by our group: uFP = log [sFlt/PlGF x 10].[5] For serum sFlt-1/PlGF ratio, the arithmetic ratio was used in calculations as previously done by others.[6]

2. Supplementary Table

2.1. Breakdown of cases with at least two triage visits based on the sequence of the Congo Red Dot (CRD) Paper Test result (n=133)

**CRD Paper Test Result** *First & Last Encounter*	**No. cases** ***n***	**Adjudicated as YES PE at Last Encounter** ***n (%)***	**MIDPE** ***n (%)***	**Gestational Weeks at Enrollment** ***median [IQR]***	**Days Enrollment to Delivery** ***median [IQR]***	**Gestational Weeks at Delivery** ***median [IQR]***
**NEG & NEG**	88	3 (3%)	14 (16%)	35 [32–36]	22 [9–38]	38 [37–39]
**NEG & POS**	22	14 (64%)	13 (59%)	33 [31–35]	12 [5–34]	36 [33–38]
**POS & POS**	19	15 (79%)	12 (63%)	31 [27–34]	7 [3–28]	33 [28–36]
**POS & NEG**	4	1 (25%)	0 (0%)	35 [33–38]	8 [2–19]	37 [35–39]

PE=preeclampsia. WP=weak positive. MIDPE=medically indicated delivery for preeclampsia. NEG=negative. POS=positive.

3. Supplementary Figures

3.1. Analytical validation of the Congo Red Dot (CRD) Paper Test against the CRD nitrocellulose array

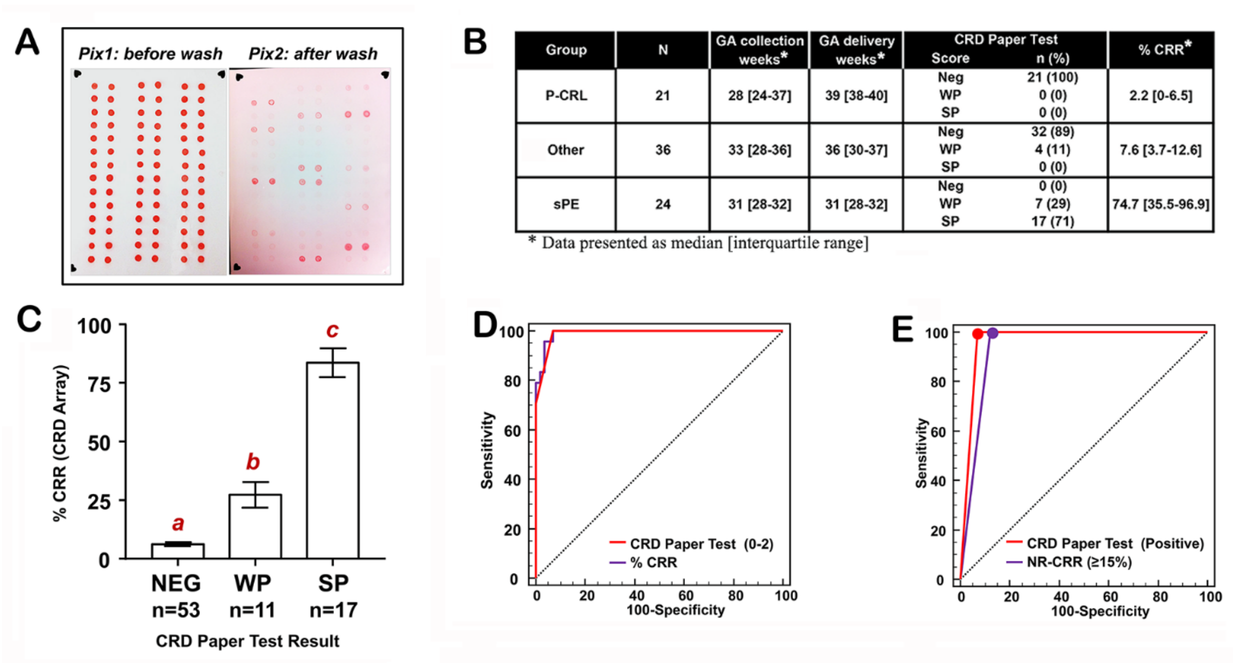

Group	N	GA collection weeks*	GA delivery weeks*	CRD Paper Test Score	n (%)	% CRR*
P-CRL	21	28 [24-37]	39 [38-40]	Neg<br>WP<br>SP	21 (100)<br>0 (0)<br>0 (0)	2.2 [0-6.5]
Other	36	33 [28-36]	36 [30-37]	Neg<br>WP<br>SP	32 (89)<br>4 (11)<br>0 (0)	7.6 [3.7-12.6]
sPE	24	31 [28-32]	31 [28-32]	Neg<br>WP<br>SP	0 (0)<br>7 (29)<br>17 (71)	74.7 [35.5-96.9]

* Data presented as median [interquartile range]

This analytical validation study used 81 urine samples retrieved from women in the following clinical groups: **1)** preeclampsia with severe features requiring medically indicated delivery (sPE, n=21); **2)** non-hypertensive women with pregnancy-related conditions unrelated to PE (Other, n=36, e.g. spontaneous preterm labour, isolated foetal growth restriction, oligohydramnios) and **3)** healthy pregnant controls (P-CRL, n=21). These samples did not belong to the clinical triage cohort participants, but to patients recruited prospectively and consecutively as part of our institutional bio-bank research initiative. Each urine sample was tested both with the CRD Paper Test kit and the CRD nitrocellulose array. **(A)** Representative photographs of a CRD nitrocellulose array before (Pix1) and after (Pix2) washing with methanol. **(B)** Distribution of CRD Paper Test scores for the 81 urine samples used for analytical validation. Gestational age (GA) at sample collection and delivery for the three groups is included. Samples from P-CRL women and the majority of samples from women with other pregnancy complications scored negative (NEG) on the CRD Paper Test. Samples from sPE women scored either strong positive (SP, 71%) or weak positive (WP, 29%). **(C)** %CRR (mean±standard error) for samples scoring NEG, WP or SP on the paper test. There was a statistically significant difference in %CRR between samples scored WP or strong SP and those that scored NEG. Columns with different letters denotes statistical significance at P<0.05. **(D)** Receiver Operating Characteristic (ROC) plots of %CRR as continuous variable and CRD Paper Test on ordinal scale 0-2 (NEG=0, WR=1, SP=2). The best cut-off that discriminated sPE cases from the rest was >15.6%, almost equivalent to our previously published cut-off (>15%).[3] There was no difference in accuracy between the two methods as judged by area under the ROC plot (AUROC) (P=1.0). **(E)** Likelihood ratio (LR) graphs comparing the CRD Paper Test dichotomised as NEG or positive (POS: WP or SP) and %CRR dichotomised as reassuring (≤15%) or non-reassuring (NR-CRR, >15%). There was no significant difference between the two methods (P=0.256). These results remained the same when the %CRR derived from urine samples tested without protein normalization. NR-CRR, non-reassuring CRR%.

3.2. Distribution of gestational age (GA) and number of triage visits

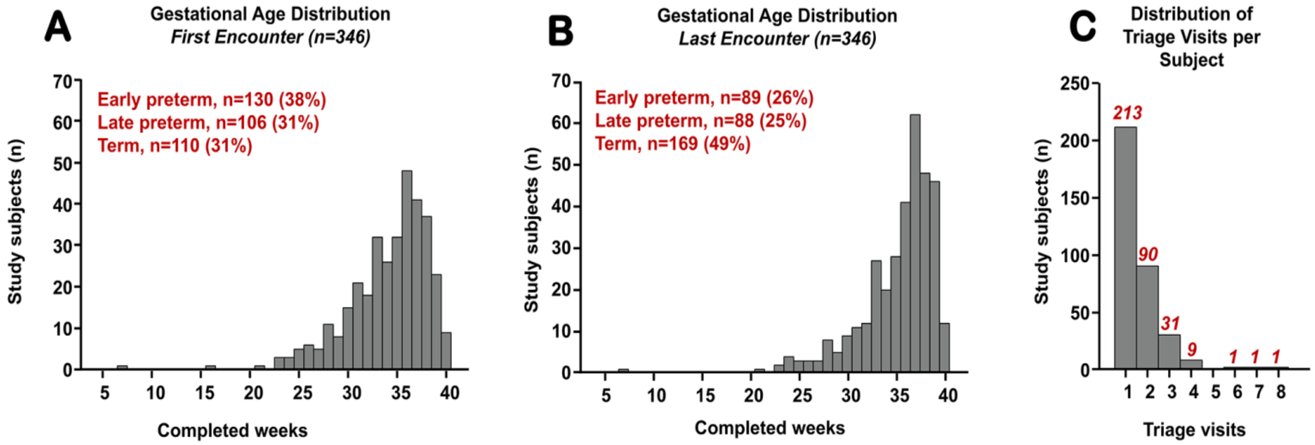

The distribution of GA at **(A)** first triage visit, **(B)** last triage visit, and **(C)** the number of triage visits per patient. Most patients presented for their first or last triage visit late pre-term (34–37 weeks). The median [interquartile range] for the first and last visit were 35 [32–37] weeks and 36 [33–38] weeks, respectively. Most patients had 1[1–2] triage visits.

3.3 Comparative likelihood ratio graphs of Congo Red Dot (CRD) Paper Test against urine and serum markers measured in the research laboratory

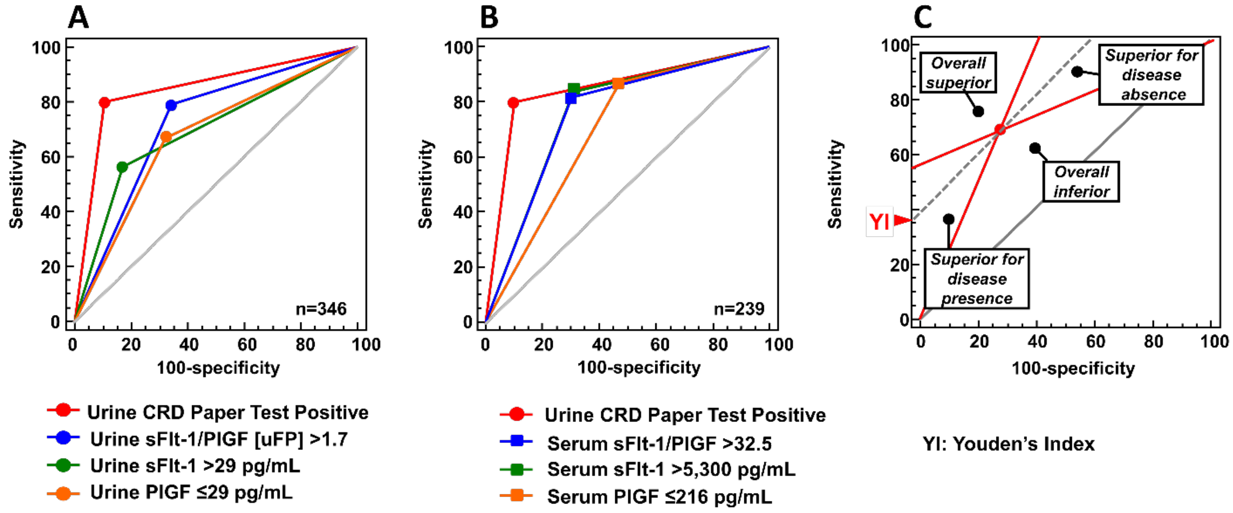

Likelihood ratio graphs comparing other **(A)** urine and **(B)** serum markers to the CRD Paper Test (index test), and **(C)** visual aid for interpretation of the results. The "reference standard" was the adjudicated diagnosis at the last triage encounter. The urine and serum markers were dichotomised based on their optimal cut-off in this population (maximal Youden index) and were considered the comparator tests.

The aid for interpretation of the results shows that the slopes (tangents) of the lines connecting the cut-off point of a test to points (0, 0) and (100, 100) represent the positive likelihood ratio (LR+) and the LR–, respectively. The LR+ and LR– lines divide the chart into four areas. Depending on the position of an index test relative to a comparator test, it can be appreciated as overall superior, overall inferior, superior for confirming the absence of the disease under investigation, or superior for confirming the presence of the same disease. Youden's Index is the intercept on the vertical axis of a line whose slope is 1, passing through the cutoff point.[7,8]

The CRD Paper test overall superior at diagnosing preeclampsia compared to serum or urine soluble fms-like tyrosine kinase-1 (sFlt-1), placental growth factor (PlGF), and sFlt-1/PlGF ratio (uFP).

3.4. Comparative likelihood ratio graph of Congo Red Dot (CRD) Paper Test against blood pressures and clinical measures of total proteinuria

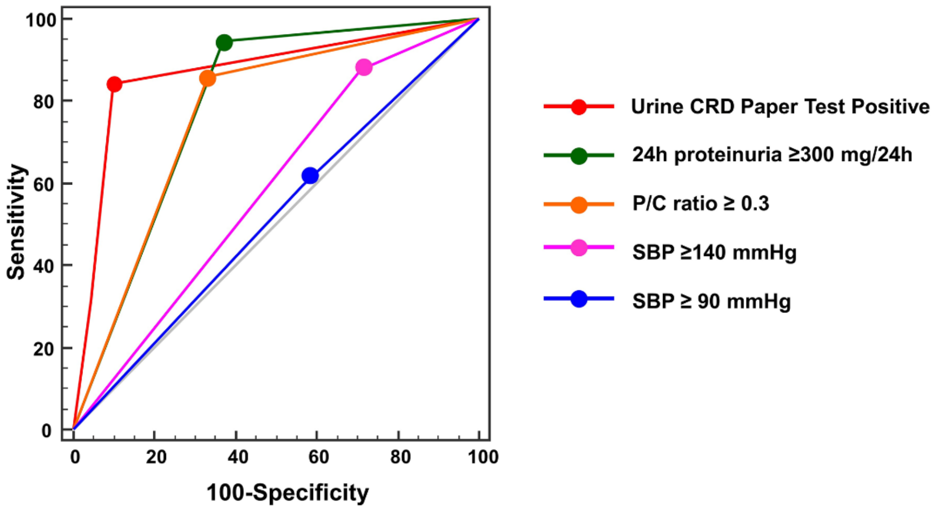

Likelihood ratio graph comparing the CRD Paper Test with blood pressures and clinical total proteinuria in the subgroup of women who completed a 24-hour proteinuria evaluation (n=168). The "reference standard" was the adjudicated diagnosis at the last triage encounter. Systolic blood pressure (SBP), diastolic blood pressure (DBP), protein-to-creatinine (P/C) ratio and 24h-proteinuria were dichotomised based on clinically relevant cut-offs and were considered the comparator tests. The CRD Paper Test had the highest area under the ROC plot (AUROC=0.868, 95%CI [0.807-0.915] compared to individual clinical variables: 24h proteinuria ≥ 300mg (0.789, 95%CI [0.179-0.848], P=0.045), P/C ratio (0.763, [0.691-0.825], P=0.005), SBP (0.583, 95%CI [0.504-0.658], P<0.001) and DBP (0.514, 95%CI [0.436-0.592], P<0.001).

3.5. Fagan plot analysis to evaluate the clinical utility of the CRD Paper Test based on pre-and post-test probabilities.

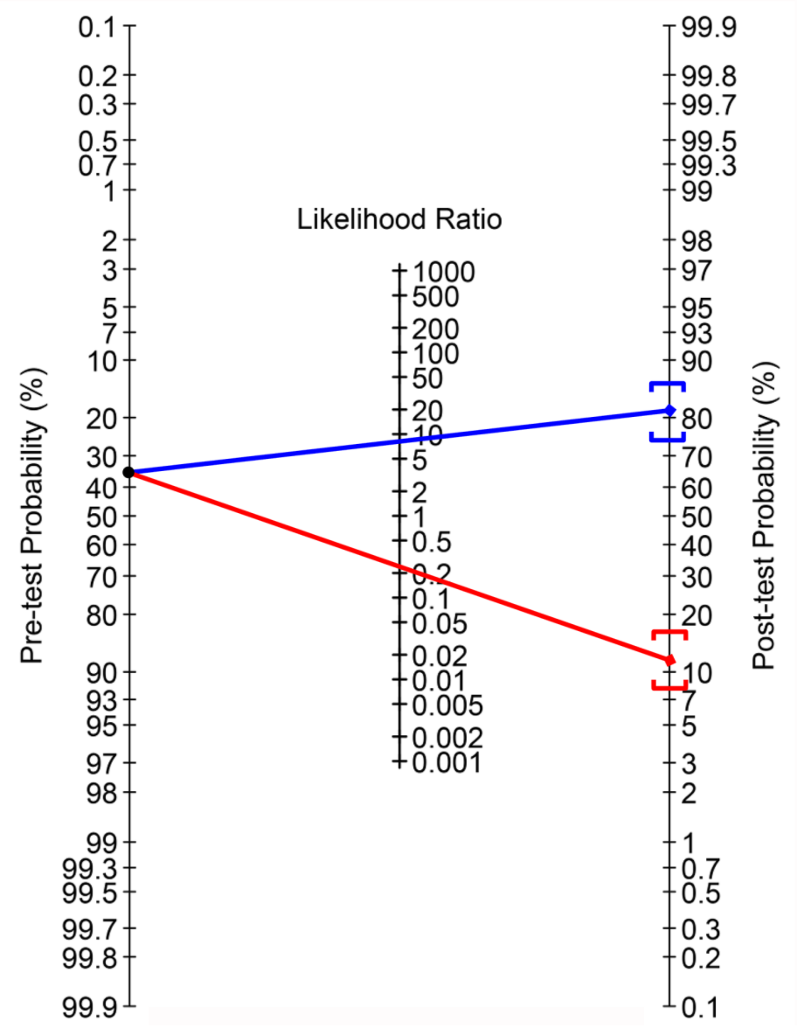

The CRD Paper test's pre-test probability of 27·7% was based on the prevalence of PE based on adjudicated diagnosis. The CRD Paper Test had a positive post-test probability of 74·0% 95%CI [66–81] and a negative post-test probability of 8·0% 95%CI [5–11]. The Fagan plot [9] consisted of a vertical axis on the left with the pre-test probability, an axis in the middle representing the likelihood ratio and a vertical axis on the right representing the post-test probability.

References

1 Buhimschi IA, Buhimschi CS, Glabe CG. Methods and compositions for the detection and treatment of preeclampsia. Non-provisional patent application claiming priority form U.S. Provisional Application No. 61/197,914, filed on October 31, 2008 and U.S. Provisional Application 61/206,534, filed January 29, 2009. Patent Number 9229009 Issued January 5, 2016.

2 Buhimschi IA, Buhimschi CS, Tagare H, Choma M, Jonas S. Methods and compositions for detecting misfolded proteins. Non-provisional patent application filed April 1, 2015 claiming priority from Provisional patent application filed April 2014 claiming priority form U.S. Provisional Application No. 61/197,914, filed on October 31, 2008 and U.S. Provisional Application 61/206,534, filed January 29, 2009.

3 Buhimschi IA, Nayeri UA, Zhao G, et al. Protein misfolding, congophilia, oligomerization, and defective amyloid processing in preeclampsia. *Sci Transl Med* 2014; **6**: 245ra92.

4 Jonas SM, Deserno TM, Buhimschi CS, Makin J, Choma MA, Buhimschi IA. Smartphone-based diagnostic for preeclampsia: an mHealth solution for administering the Congo Red Dot (CRD) test in settings with limited resources. *J Am Med Inform Assoc* 2016; **23**: 166–73.

5 Buhimschi CS, Norwitz ER, Funai E, et al. Urinary angiogenic factors cluster hypertensive disorders and identify women with severe preeclampsia. *Am J Obstet Gynecol* 2005; **192**: 734–41.

6 Signore C, Mills JL, Qian C, Yu K, Lam C, Epstein FH, Karumanchi SA, Levine RJ. Circulating angiogenic factors and placental abruption. *Obstet Gynecol* 2006; **108**: 338–44.

7 Mastrovitch TA, Bithoney WG, DeBari VA, Nina AG. Point-of-care testing for drugs of abuse in an urban emergency department. *Ann Clin Lab Sci* 2002; **32**: 383–6.

8 Youden WJ. Index for rating diagnostic tests. *Cancer* 1950; **3**: 3–35.

9 Fagan TJ. Letter: nomogram for Bayes' theorem. *N Engl J Med* 1975; **293**: 257.
